# Electric Pulses Can Influence Galvanotaxis of* Dictyostelium discoideum*

**DOI:** 10.1155/2018/2534625

**Published:** 2018-08-08

**Authors:** Ying Li, Yu Gu, He Wang, Zhipeng Liu, Bing Song, Tao Yin

**Affiliations:** ^1^Chinese Academy of Medical Sciences & Peking Union Medical College Institute of Biomedical Engineering, Tianjin, China; ^2^Cardiff Institute of Tissue Engineering & Repair, School of Dentistry, College of Biomedical & Life Sciences, Cardiff University, Cardiff, UK

## Abstract

Galvanotaxis, or electrotaxis, plays an essential role in wound healing, embryogenesis, and nerve regeneration. Up until now great efforts have been made to identify the underlying mechanism related to galvanotaxis in various cells under direct current electric field (DCEF) in laboratory studies. However, abundant clinical research shows that non-DCEFs including monopolar or bipolar electric field may also contribute to wound healing and regeneration, although the mechanism remains elusive. Here, we designed a novel electric stimulator and applied DCEF, pulsed DCEF (pDCEF), and bipolar pulse electric field (bpEF) to the cells of* Dictyostelium discoideum*. The cells had better directional performance under asymmetric 90% duty cycle pDCEF and 80% duty cycle bpEF compared to DCEF, with 10 Hz frequency electric fields eliciting a better cell response than 5 Hz. Interestingly, electrically neutral 50% duty cycle bpEF triggered the highest migration speed, albeit in random directions. The results suggest that electric pulses are vital to galvanotaxis and non-DCEF is promising in both basic and clinical researches.

## 1. Introduction

Bioelectricity is the assembly of the endogenous electric current in living organisms, from ion channels, pumps, and electrical synapses, which forms bioelectric potentials at different levels in the organisms: the nuclear envelope potential at the organelle level, the transmembrane potential at the cell level, the transepithelial potential (TEP) at the tissue level, and the major body axes at the organism level [1]. In 1794, Galvani showed that the muscle of a frog's leg could be stimulated by the cut terminal of the sciatic nerve of its counterlateral leg, which indicated the existence of the injury potential as well as the motor potential. However, the therapeutic effect of the bioelectricity had not been considered as “scientific” by the mainstream scientists, and the studies of the injury potential had made little progress for over a century due to the lack of precise and handy instruments and the abuse of the therapeutic electric stimulation carried out by charlatans without serious investigation [2], compared with the fast development of the motor neuron potential in the field of electrophysiology. In 1970s, along with the invention of a novel vibrating probe [3], new research on injury potentials commenced, and the galvanotaxis of the cells was discovered. Injury current and endogenous electric field (later known as TEP) were detected in the stump of newt limbs [4] and the skin of guinea pig [5] and humans [6]. Subsequently, transcorneal potential difference (TCP) was found in the bovine eyes [7] and the eyes of rats [8] and humans [9]. The finding of TEP and TCP led researchers to believe that cells can sense and migrate along the electric cues; human keratinocytes reoriented and migrated towards cathode in cell culture under a small electric field [10]. In cell culture, various cells were found to migrate directionally in a physiological strength DCEF [11], which is called galvanotaxis or electrotaxis. Furthermore, regulating the endogenous potentials affected the wound healing rate as was shown in a study on TCP using pharmacological agents [12]. Exposure to magnetic fields was found to induce behavior and different migratory responses in various cells, with some of the underlying mechanisms elucidated on in* in vitro* studies; postganglionic sympathetic neurons (PSNs) and neural crest-derived neurons were found to align perpendicular to physiological voltage [13, 14], while neural precursor cells were found to migrate towards cathode in DCEF [15]. In endothelial cells, the MAPK/ERK pathways were regulated by DCEF [16]. Lung adenocarcinoma cells were found to migrate towards anode independent of serum and EGFR (epidermal growth factor receptor) [17]. The epithelial sodium channel was found to mediate galvanotaxis without affecting migration speed in human keratinocytes [18]. An electrochemical pathway was found in budding yeast to orient the cell polarization [19]. Activation of focal adhesion kinase (FAK) signaling was found to be vital to the motility of trophoblast cells in EFs [20]. Human mesenchymal stem cells were found to migrate towards cathode depending on the EF strength [21]. Angiogenesis as well as wound healing was induced by DCEF in wound healing model of human skin [22]. ATP-mediated mechanism was found in galvanotaxis of keratinocyte [23]. Voltage-gated Kv1.2 channels were found to be important in galvanotactic response of COS-7 cells [24]. Pax6 was found required for cell galvanotaxis in cortical neurospheres [25].

Based on the current knowledge, galvanotaxis, or electrotaxis, plays an essential role in wound healing, embryogenesis, and nerve regeneration. The galvanotaxis of various cells under DCEF has been investigated, and the possible mechanisms [26–29] and quantitative relations between DCEF dosage and galvanotactic response efficiency have been elucidated [30, 31]. Notwithstanding the findings of the laboratory studies, clinical research has shown promising results on wound healing and regeneration using non-DCEF stimulation [32]. However, the galvanotaxis under non-DCEF still remains elusive. In this study, we investigated the similarities and differences in the behavior of cells under non-DCEF and DCEF. Here, we designed a novel electric stimulator capable of applying DCEF, pDCEF, and bpEF (both symmetric and asymmetric) in a galvanotactic chamber to elucidate the characteristics of the galvanotaxis of* D. discoideum*.

*Dictyostelium discoideum* is used as a model cell for cell motility, chemotaxis, signal transduction, and cell differentiation during development [33–35]. It undergoes changes from single cell to multicellular system in its life cycle. The signaling pathways underpinning galvanotaxis of* D. discoideum* are PI3K and PTEN [26, 28], which are partly shared with that of chemotaxis. The threshold of the galvanotaxis of amoeba has been reported as being around 1 V/cm, with the directedness reaching 50% of its maximum value at 2.6 V/cm [30]. Using* D. discoideum* as a model, we can predict the behaviors of the cells involved in wound healing, such as keratinocytes, fibroblast, and lymphocytes based on the findings from the* D. discoideum* study [26, 33, 34].

In this study, we applied various specifications for the electric fields: the frequencies used to treat the cells were 5 and 10 Hz, the duty cycle settings were 50% and 90% in pDCEF and 50%, 80%, and 90% in bpEF; the polarity was unipolar for pDCEF and bipolar for bpEF. Furthermore, we introduced a novel parameter, EMR (effective migration ratio), for the assessment of cell behavior in electric fields, based on actual trajectory of the cell, which is especially useful in discrimination of two groups of cells in the same level of directedness.

## 2. Methods

### 2.1. Cell Preparation

The cells of* D. discoideum*, AX2 (wild type), were grown in a Petri dish with HL5 (Formedium, UK) in medical cooler at 21°C. When approaching ~80% subconfluency, the cells were transferred into a flask with HL5 Glucose (ibid) on the shaker (KS-130, IKA Co., Germany) for mass culture. When a sufficient number of cells are achieved, the cells were washed by development buffer (DB, 1.34 g/L of Na_2_HPO_4_ × 7H_2_O, 0.68 g/L of KH_2_PO_4_, 2 mM MgSO_4_, and 0.2 mM CaCl_2_) and counted to make the density 1~2 × 10^6^ cells/ml before starvation. They were transferred into a flask with DB on the shaker, starved for 1 hour, treated with 0.1 ml cAMP every 6 min for 4 hours using the peristaltic pump (MINIPULS 3, Gilson Inc.), and then washed by DB before time-lapse photography.

### 2.2. Cell Migration Analysis

**Figure 1:**
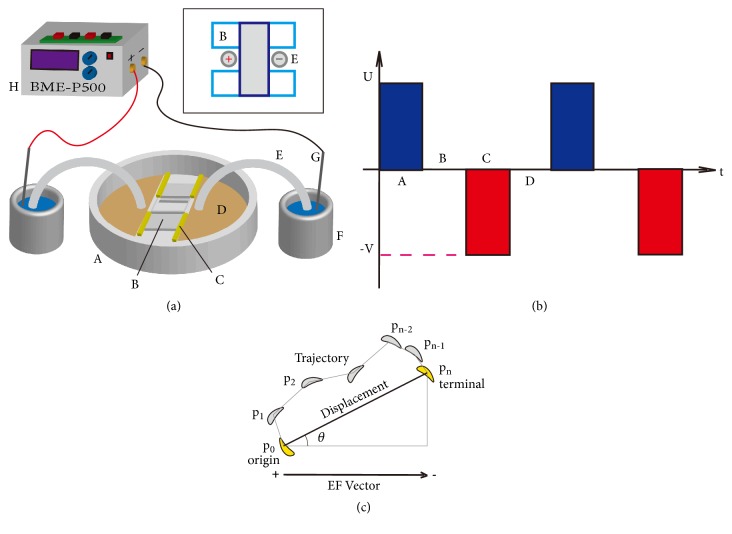
The schematic of the galvanotactic chamber and the electric stimulator, the composition of the electrical waveforms, and the analysis of cell motility in galvanotactic image sequence. (a) The galvanotactic chamber and the stimulator schematics consists of 8 items: (A) the 10-cm Petri dish, (B) the coverslip bridge to observe the cells and let the medium flow, (C) the silicon grease walls to build reservoir, (D) the reservoir to store medium, (E) a pair of agar bridges to connect buffer beakers to the chamber, (F) the buffer beakers to store Steinberg's solution, (G) Ag/AgCl electrodes to apply electricity, and (H) the stimulator to generate DCEF, pDCEFs, and bpEFs. Inset: top view of the coverslip (B) bridge and agar bridges (E). (b) The variable composition of the electric waveforms: positive stimulation with amplitude of U (A), zero stimulation after positive stimulation (B), negative stimulation with amplitude of -V (C), and zero stimulation after negative stimulation (D). (c) The analysis of cell motility in image sequence: the original position of the cell is P_0_ in the first frame, P_1_ in the second frame, and the terminal position P_n_ in the last frame. The EF vector is from anode pointing to the cathode under DCEFs and pDCEFs, or the “net anode” pointing to the “net cathode” under bpEFs. The displacement of the cell is from P_0_ to P_n_, and its vector is P_0_ pointing to P_n_. The angle of the cell *θ* is between the vectors of the EF and its displacement. The trajectory of the cell is algebraic sum of all the displacement between two successive frames.

Directedness (cos⁡*θ*) is a parameter to indicate the degree of the directional tendency of the cell migrating along with the direction of the electric field, as in the previous study [36]. As shown in Figure 1(c), angle *θ* of every cell is from the direction of the electric field to the displacement vector which is the original position of the cell in the first frame pointing to the terminal position in the last frame. Given that the coordinates of the origin and terminal are P_0_ (X_0_, Y_0_) and P_n_ (X_n_, Y_n_), respectively, the directedness is

(1) $$\begin{array}{c} \cos\theta =\frac{X_{n}-X_{0}}{\sqrt{{\left(X_{n}-X_{0}\right)}^{2}+{\left(Y_{n}-Y_{0}\right)}^{2}}} \end{array}$$

The cosine of the angle is the practical index for directedness: positive value indicates that the cell migrates towards the cathode and negative value towards the anode, and the cell with absolute value greater than 0.707 indicates that it migrates more along with the electric field than being perpendicular to it. The average of directedness reveals the overall directional tendency of the cells as a whole. Total trajectory (S_Total_, in *μ*m) is a parameter to indicate the cell motility which is the algebraic sum of each displacement of the cell between two successive frames. Given that the coordinates of all the cells are P_0_ (X_0_, Y_0_), P_1_ (X_1_, Y_1_),…, P_n_ (X_n_, Y_n_), respectively, accordingly, the displacement from the frame 0 to frame 1 is D_1,0_, the displacement from frame 2 to frame 1 is D_2,1_,…, and the displacement from frame n-1 to frame n is D_n,n-1_. Hence, the total trajectory is

(2) STotal=∑n=1nDn,n−1=X1−X02+Y1−Y02+⋯+Xn−Xn−12+Yn−Yn−12

and, furthermore, trajectory speed (V_cell_, in *μ*m/min) is total trajectory of the cell (in *μ*m) divided by its total migration time (in min):

(3) $$\begin{array}{c} V_{cell}=\frac{S_{Total}}{t} \end{array}$$

EMR which we have introduced in this article is the ratio of the x-axis component of the displacement of the cell to its total trajectory, which is a more precise measure than the directedness for revealing the percentage of the cell's migration along the electric field to its actual route and especially useful for analyzing cell groups with similar directedness but different trajectory pattern:

(4) $$\begin{array}{c} EMR=\frac{X_{n}-X_{0}}{S_{Total}}=\frac{X_{n}-X_{0}}{\sqrt{{\left(X_{1}-X_{0}\right)}^{2}+{\left(Y_{1}-Y_{0}\right)}^{2}}+\cdots +\sqrt{{\left(X_{n}-X_{n-1}\right)}^{2}+{\left(Y_{n}-Y_{n-1}\right)}^{2}}} \end{array}$$

The average of the EMR of all the cells indicates the overall directional tendency of the cell group.

### 2.3. Galvanotaxis Experiment Setups and Data Analysis

The galvanotaxis experiment consists of 3 main parts: the galvanotactic chamber with buffer beakers, a user-designed electrical stimulator, and an imaging system including a Zeiss Axiovert 100 microscope with 10× objective lens, CoolSNAP HQ camera system (Photometrics, US), and MetaMorph imaging system and a motorized stage (Universal Imaging, US) used to record time-lapse images of the cells in various positions in the observed area. As in the previous studies [36, 37], and shown in Figure 1(a), to prepare the galvanotactic chamber in 10-cm Petri dish (A), three coverslips (B) were cut in half to make 10×20×0.1 mm^3^ pieces. Two pieces (light blue in inset) were aligned in parallel as foundation of the bridge with a distance of 10 mm and 1 piece (blue in inset) as the roof. The width of the roof coverslip determined the distance of all the electric fields we applied, which was 10 mm. In the observed area, the applied electric field was regarded as homogenous due to its relatively small dimension. Silicon grease (DC4, Dow Corning, UK) was used to glue the pieces together and build blocking walls (C) from the sides of the bridge to the sides of the Petri dish and make up two separated reservoirs (D) connected by the bridge. The targeted cells were injected into the center of the bridge tunnel before the roof coverslip was placed onto the bridge-foundation coverslips, and finally DB whose pH was 7.4 was injected as reservoir medium in the chamber. A pair of salt bridges with the gel of 1% agar in DB (E) was placed in the vicinity of both sides of the bridge (inset) to make electrical connections between the chamber and the buffer beakers (F), and, therefore, the distance between agar bridges in the chamber was 10 mm, which was the distance of all the applied electric fields in this article. Steinberg's solution (58 mM NaCl, 0.67 mM KCl, 0.44 mM Ca(NO_3_)_2_×4H_2_O, 1.3 mM MgSO_4_×7H_2_O, and 4.6 mM TrismaBase) was used as buffer medium in the buffer beakers. An electrical stimulator (H) capable of supplying DCEF, pDCEFs, and bpEFs was designed and connected to the beakers via Ag/AgCl electrodes (G) and the wires to impose the electric stimulation onto the cells in the chamber. The left electrode of the chamber was connected to the positive electrode of the stimulator when applying 10-Hz waveforms and to the negative one 5-Hz waveforms. All the voltage waveforms of the chamber and stimulator were monitored and recorded by the oscilloscope (TENMA 72-10510, Farnell, UK).

The cells were injected to the chamber for 20 mins before time-lapse photography. Time-lapse pictures were recorded with time interval of 20 seconds between two successive frames for over 30 mins or more. The time-lapse sequences were tracked by ImageJ software with Chemotaxis Toolkit, and the data were analyzed in Excel (Microsoft, US) and Prism (GraphPad Software, US) using one-way and two-way ANOVA (analysis of variance) in which Tukey's multiple comparison test was used in the comparison of every two datasets of different conditions.

### 2.4. Design of the Electric Stimulator

**Figure 2:**
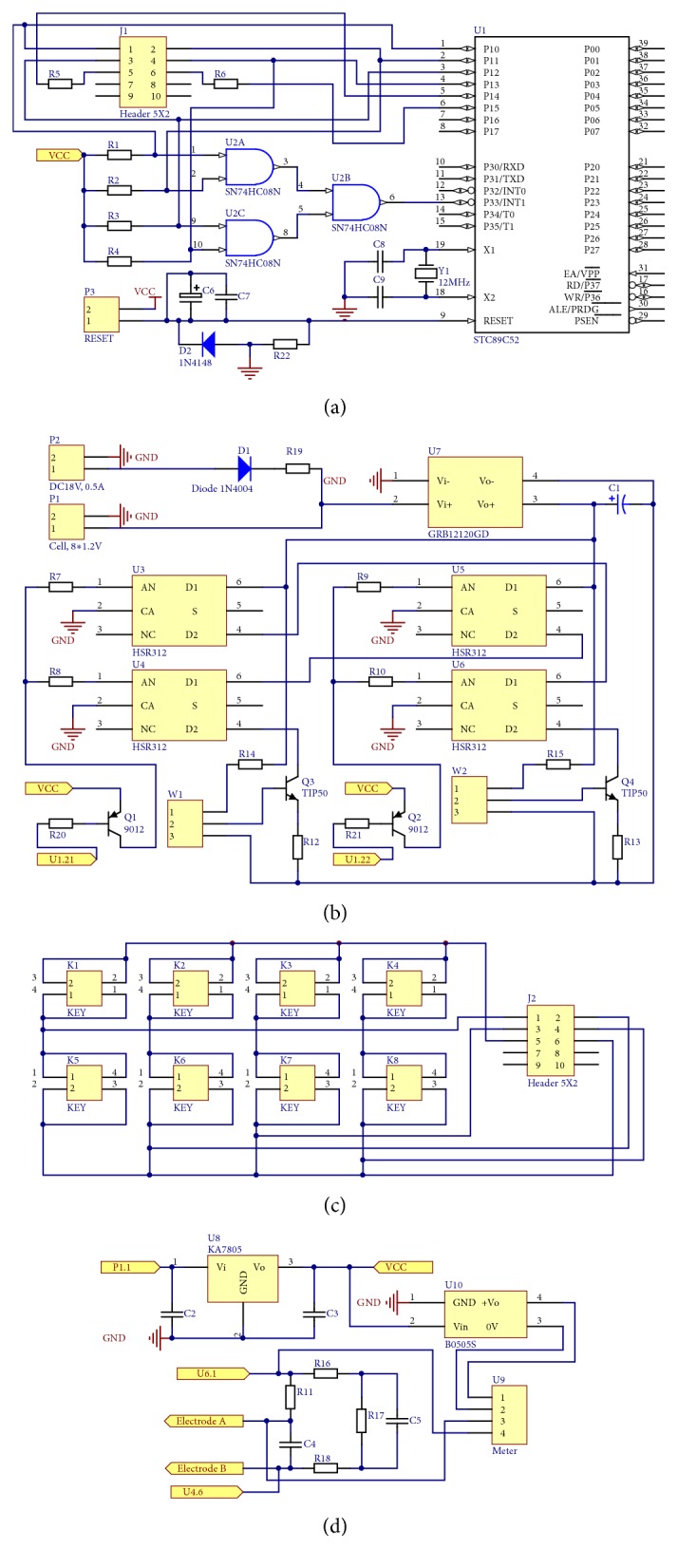
The schematic of 4 units of the stimulator. (a) The microcontroller unit with reset and keypad decoder circuits, (b) the high-power output unit with rechargeable battery, high-voltage DC/DC, H-bridge, and voltage/current adjusting circuit, (c) the keypad unit, and (d) the monitor unit with output electrodes.

The device BME-P500 we designed was an electric stimulator, whose voltage was 120 V maximum (240 V peak to peak in bipolar mode), current was 10 mA maximum, frequency was 500 Hz maximum, duty cycle was adjustable, and waveforms could be adjusted to DCEF, pDCEF, or bpEF. The device consisted of 4 parts: the microcontroller unit which was to receive commands from users and to regulate the on-off states of the optocouplers, the high-power output unit to supply high-voltage electrical stimulation, the keypad unit to respond to the user's input, and the monitor unit to show the instant current value, as shown in Figure 2.

In the microcontroller unit, as shown in Figure 2(a), U1 was the MCU (microcontroller unit) component (STC89C52, STC Micro, China) compatible with the MCU-51 architecture; the crystal oscillator Y1 (12 MHz, quartz crystal) and the capacitors C8 and C9 (30 pF, ceramic, all capacitors used were of 25-V level, otherwise specified) made up the crystal circuit for MCU; the pushbutton P3, the capacitors C6 (22 *μ*F, electrolytic) and C7 (0.1 *μ*F, ceramic), the diode D2 (1N4148), and the resistor R22 (10 kΩ, metal film, all the resistors used were of 1/8 watt, otherwise specified) made up the reset circuit; the AND gates U2 (SN74LS08N, TI instruments, US), the resistors R1-R4 (4.7 kΩ, metal film) and R5-R6 (150 Ω, metal film), and the connector J1 (Header 5 × 2) made up the on-board keypad decoder circuit.

In the high-power output unit, as shown in Figure 2(b), the connectors P1 (for 18 V, 0.5 A direct current power supply from power plug) and P2 (for 8 × 1.2 V-rechargeable-cell case), the diode D1 (1N4004), and the resistor R19 (47 Ω-5 W, cement) made up the battery-recharging circuit to store and supply electricity to all the circuits; the high-voltage DC/DC (direct current to direct current converter) U7 (GRB12120GD, Shenzhen Aotong Technology Co., China) and its coupling capacitor C1 (22 *μ*F, electrolytic-250 V) were used to supply high voltage to the power part; the optocoupler U3-U6 (HSR312, Fairchild Semiconductor, US), the transistors Q1 and Q2 (SS9012, Fairchild Semiconductor, US), the power transistors Q3 and Q4 (TIP50, STMicro, Italy), the resistors R7-R10 (330 Ω, metal film), the resistors R14-R15 (680 kΩ, metal film), the resistors R20-R21 (10 kΩ, metal film), and the resistors R12-R13 (100 Ω-1 W, carbon film) made up the H-bridge power output circuit with the trimmers W1 and W2 (100 kΩ – 2W, Bochen, China) capable of adjusting the output current along with the output voltage.

In the keypad unit, as shown in Figure 2(c), the pushbuttons K1-K8 made up the key matrix, and the connector J2 (Header 5 × 2) was the counterpart of J1 in MCU unit.

In the monitor unit, as shown in Figure 2(d), the regulator U8 (KA7805, Fairchild Semiconductor, US) and the capacitors C2 and C3 (0.1 *μ*F, ceramic) were used to supply operation voltage (nominal VCC, +5 V); the isolated regulator U10 (B0505S, Mornsun Co., China) and the meter component U9 (ZX5135B-DV2V, Zhengxie Meters, China), the resistors R11 (100 Ω – 1 W, carbon film), R16 (470 kΩ, metal film), R17 (47 kΩ, metal film), and R18 (470 kΩ, metal film) and the capacitors C4 (5 nF – 500 V, film) and C5 (5 nF – 50 V, film) were used to monitor the current of output circuit. The electrodes A (positive) and B (negative) were connected to the two terminals of the capacitor C4.

### 2.5. Design of Stimulating Waveforms

The Keil uVision software (ARM, US) was used to program the C51 code and interpret the code into machine code and the STC-ISP software (STC In-System Programming, STC Micro, China) was used to download the machine code to the microcontroller. In this program, we defined 10 milliseconds as the period of the timer, upon which we set the on-off state of optocoupler to construct different waveforms. All the waveforms were constructed by 4 parts in a single period, i.e., parts A, B, C, and D (optional), as shown in Figure 1(b), which were the positive stimulation of the period with the amplitude of U, the zero-voltage output after positive pulse, the negative pulse with the amplitude of V, and the zero-voltage output after negative pulse, respectively. Using different length of these 4 parts, 10 different types of the electric waveforms were constructed, as shown in Table 1; e.g., “10 Hz Uni 50%” means the frequency is 10 Hz, the polarity is unipolar, i.e., pDCEF, and the duty cycle is 50%, whereas “5 Hz Bip 80%” means the frequency is 5 Hz, the polarity is bipolar, i.e., bpEF, and the duty cycle is 80%.

## 3. Results

**Figure 3:**
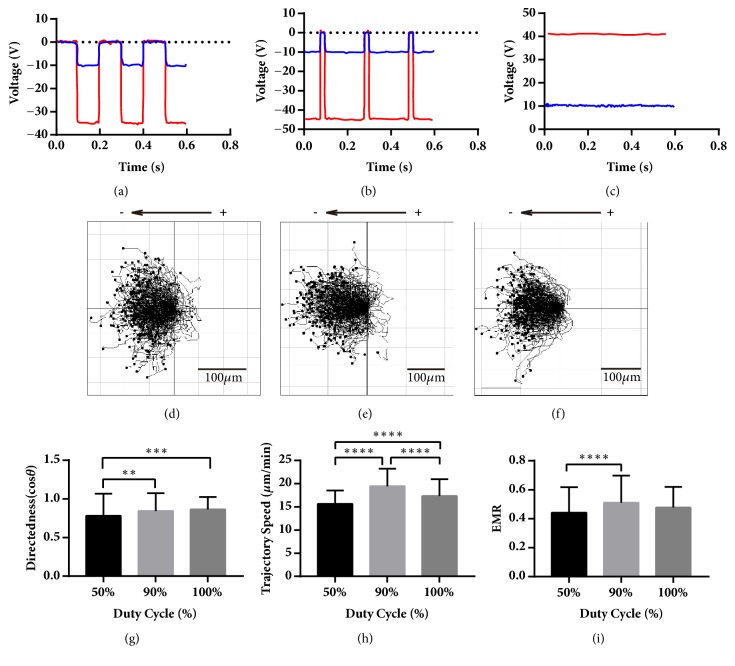
The waveforms of the chamber and the stimulator, the collective galvanotaxis trajectory, and the analysis of the directedness, trajectory speed, and EMR under 5 Hz pDCEF with duty cycle of 50% and 90%, and 10V/cm DCEF. (a-c) The waveforms of the chamber (blue) and the stimulator (red) for 5 Hz 50% duty cycle pDCEF, 5 Hz 90% duty cycle pDCEF, and 10 V/cm DCEF. (d-f) The galvanotactic trajectory of all 240 cells under 5 Hz 50% duty cycle pDCEF, 5 Hz 90% duty cycle pDCEF, and 10 V/cm DCEF. They all migrated towards the “net cathodes”. (g) The cells under 5 Hz 50% pDCEF was lower than 90% pDCEF and 10V/cm DCEF in directedness. ANOVA on different duty cycle: *∗∗∗* p < 0.001. For multiple comparisons, 50% versus 90%: *∗∗* p < 0.01; 50% versus 100% (DCEF): *∗∗∗* p < 0.001. (h) The cells under 5 Hz 90% pDCEF migrated the fastest, and 50% pDCEF the slowest. ANOVA on different duty cycle: *∗∗∗∗* p < 0.0001. For multiple comparisons, 50% versus 90%: *∗∗∗∗* p < 0.0001; 90% versus 100%: *∗∗∗∗* p < 0.0001; 50% versus 100% (DCEF): *∗∗∗∗* p < 0.0001. (i) The cells under 90% pDCEF have better EMR than that of 50%. ANOVA on different duty cycle: *∗∗∗∗* p < 0.0001. For multiple comparisons, 50% versus 90%: *∗∗∗∗* p < 0.0001. One-way ANOVA and Tukey's test of multiple comparisons were used.

**Figure 4:**
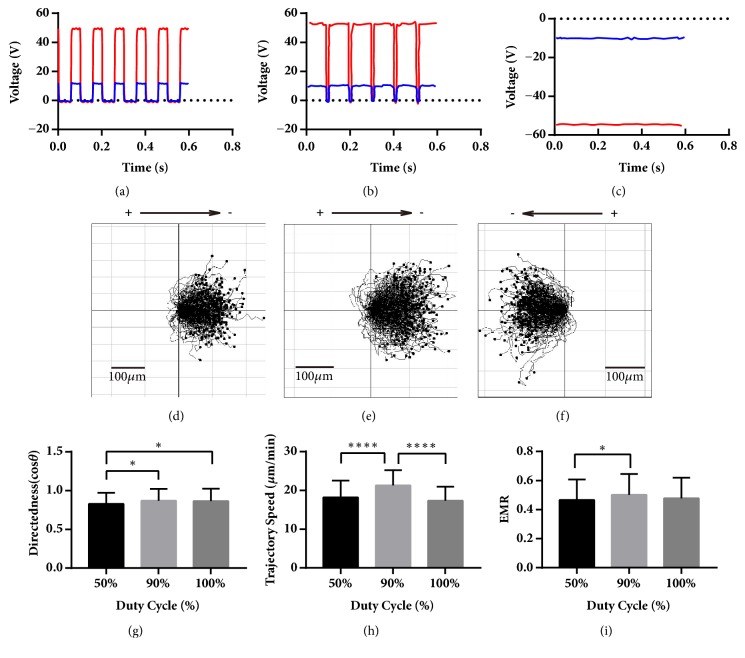
The waveforms of the chamber and the stimulator, the collective galvanotaxis trajectory, and the analysis of the directedness, trajectory speed, and EMR under 10 Hz pDCEF of 50% and 90%, and 10 V/cm DCEF. (a-c) The waveforms of the chamber (blue) and the stimulator (red) for 10 Hz 50% duty cycle pDCEF, 10 Hz 90% duty cycle pDCEF, and DCEF. (d-f) The galvanotactic trajectory of all 240 cells under 10 Hz 50% duty cycle pDCEF, 10 Hz 90% duty cycle pDCEF, and DCEF. All the cells migrated towards the “net cathodes”. (g) The cells under 10 Hz 50% pDCEF were lower than 90% pDCEF and DCEF in directedness. ANOVA on different duty cycle: *∗∗* p < 0.01. For multiple comparisons, 50% versus 90%: *∗* p < 0.05; 50% versus 100% (DCEF): *∗* p < 0.05. (h) The cells under 10 Hz 90% pDCEF migrated the fastest. ANOVA on different duty cycle: *∗∗∗∗* p < 0.0001. For multiple comparisons, 50% versus 90%: *∗∗∗∗* p < 0.0001; 90% versus 100% (DCEF): *∗∗∗∗* p < 0.0001. (i) The cells under 90% pDCEF had better EMR than that under 50%. ANOVA on different duty cycle: *∗* p < 0.05. For multiple comparisons, 50% versus 90%: *∗* p < 0.05. One-way ANOVA and Tukey's test of multiple comparisons were used.

Under no electric field, we observed the cells with directness of 0.04 ± 0.69, n = 240; trajectory speed of 11.98 ± 3.63, n = 240, as in a previous study [30, 38]; EMR of 0.03 ± 0.33, n = 240, which was distinct regarding the cells' behavior under electric field, except that it induced similar directional characteristics of the cells in the electrically neutral (50% duty cycle) bpEF which will be detailed later. The resistance of chamber was about 26 to 30 kΩ, lower than 200 kΩ produced by another method [30], and the salt bridges were about 47 to 51 kΩ and 50 to 55 kΩ, respectively. Therefore, the ratio of the resistance of the chamber to the agar bridges was about 1:3 (30 kΩ and 97 kΩ) to 1:4 (26 kΩ and106 kΩ), which means when applying electric field, the voltage across the chamber would be 1/3 of that across agar bridges. When applying 10 V/cm DCEF on the chamber, the voltage drop across agar bridges was 40 V DC (Figure 3(c)) and 55 V DC (Figure 4(c)), respectively. We can deduce the EF simply from the applied voltage and the distance across the cells between the electrodes, because the observed area is small enough so that we can assume that the EF around the cells is homogenous. According to Ohm's Law,

(5) $$\begin{array}{c} R=\rho \frac{l}{A}, \end{array}$$

we measured in our system R = 26 kΩ to 30 kΩ, l is fixed to 1 cm, A is fixed to 1 mm^2^, therefore the resistivity (*ρ*) = 2.6 to 3.0 Ω·m, and resultant conductivity (*σ* = 1/*ρ*) is 0.333 to 0.385 S/m.

### 3.1. Cells under 90% pDCEF Have Better Performance than 50% pDCEF in All Aspects and Better Trajectory Speed than DCEF

To determine whether the cells have similar migration characteristics under pDCEF to that under DCEF, 5 Hz 50% duty cycle pulsed DC waveform, 5 Hz 90% duty cycle pulsed DC and 10 V DC (Figures 3(a), 3(b), and 3(c)) were applied onto the chamber. The voltage amplitudes of the stimulator (in red line) were all adjusted to set the voltage amplitudes in the chamber (in blue line) to 10 V onto the coverslip bridge that simultaneously establish a 10-V/cm EF in the chamber. To achieve this, the amplitudes of the stimulator voltage were slightly different, about 35 V, 45 V, and 40 V in 50% pulsed, 90% pulsed, and DC, respectively, due to variety of the impedance of different chambers and agar bridges. In addition, when applying 10 V DC to the chamber, the voltage needed in the electric stimulator was about 40 V DC, which indicates that both the resistance and voltage of the agar bridges were about 3-fold those of the chamber. The average current was about 0.4 mA along the circuit loop (Figure 1(a)) since we measured 40 mV voltage across the monitor resistor R11 that was 100 Ω (Figure 2(d)). The period of 5 Hz waveforms was 200 ms - 100 ms of 10 V/cm EF and 100 ms of zero stimulation for 50% pDCEF (Figure 3(a)), 180 ms of V/cm EF, and 20 ms of zero stimulation for 90% pDCEF (Figure 3(b)), as in Table 1. Cells moved towards the “net cathode” which was determined by the net charge in a single period and indicated by arrow (Figures 3(d), 3(e), and 3(f)). We set the left electrode of the chamber to be positive and the accordant reference direction towards right, and the cells still regarded it as the cathode and moved towards the left (Figure 3(d)) because the waveform was negative (Figure 3(a)). In addition, knowing that the overall negative value of the directedness and EMR in the first 2 conditions was due to electrically negative settings, not the cells intrinsically moving towards anode, we inversed the sign of the directedness and EMR of every single cell in negative setting condition to make comparisons between their directional ability towards the “net cathode”. All the cells in these 3 conditions migrated towards the cathode with directedness of 0.78 ± 0.29, n = 240; 0.84 ± 0.23, n = 240; and 0.86 ± 0.16, n = 240, respectively (Figure 3(g)), where the directedness under 10 V/cm DCEF agrees with the previous studies [30, 38]. There was significant variation (P < 0.001) among the cells with different duty cycle. The directedness of the cells under 50% duty cycle pDCEF was lower than that under 90% pDCEF (P < 0.01) and DCEF (P < 0.001) and that of DCEF had a higher mean than 90% pDCEF. The trajectory speed of the cells in these 3 conditions were 15.63 ± 2.91, n = 240; 19.45 ± 3.79, n = 240; 17.36 ± 3.60, n = 240, respectively (Figure 3(h)), with 10-V/cm DCEF results being in agreement with a previous study [30]. There was significant variation (P < 0.0001) in terms of the duty cycle. The cell speed under 50% pDCEF was lower than that in 90% pDCEF (P < 0.0001) and DCEF (P < 0.0001) and that of 90% pDCEF was higher than DCEF (P < 0.0001). The EMR of the cells in these 3 conditions was 0.44 ± 0.18, n = 240; 0.51 ± 0.19, n=240; 0.48 ± 0.14, n = 240, respectively (Figure 3(i)). There was significant variation (P < 0.0001) in terms of the duty cycle. The EMR in 50% pDCEF was lower than that in 90% (P < 0.0001).

The cells were also treated with 10 Hz, 50%, and 90% duty cycle pDCEF, and, compared with those under 10 V/cm DCEF, their stimulator voltage amplitudes were about 50 V, 50 V, and 55 V, respectively; the amplitude of the chamber voltage was 10 V which simultaneously establishes a 10-V/cm EF in the chamber (Figures 4(a), 4(b), and 4(c)). The period of 10 Hz waveforms was 100 ms - 50 ms of 10 V/cm EF and 50 ms zero for 50% pDCEF (Figure 4(a)) and 90 ms of 10 V/cm EF and 10 ms of zero for 90% pDCEF (Figure 4(b)), as in Table 1. Both cells under 10 Hz 50% and 90% pDCEF moved towards the “net cathode” (Figures 4(d) and 4(e)) with directedness of 0.83 ± 0.14, n = 240; 0.87 ± 0.23, n = 240, respectively (Figure 4(g)). There was significant variation (P < 0.01) among the cells in terms of the duty cycle. The cells under 50% pDCEF were lower than that of 90% pDCEF (P < 0.05) and DCEF (P < 0.05), and the mean of 90% pDCEF and DCEF was almost the same in terms of the directedness. The trajectory speed of the cells in the 50% and 90% conditions were 18.20 ± 4.35, n = 240; 21.29 ± 3.91, n = 240, respectively (Figure 4(h)). They had significant variation (P < 0.0001) compared with those of 10 V/cm DCEF in terms of duty cycle. The cells under 90% pDCEF migrated quicker than those under 50% pDCEF (P < 0.0001) and DCEF (P < 0.0001). The EMR of the cells under 50% and 90% conditions were 0.47 ± 0.14, n = 240; 0.50 ± 0.14, n = 240, respectively (Figure 4(i)), and they had significant variation (P < 0.05) in terms of duty cycle compared with those under DCEF.

### 3.2. Cells under 80% bpEF Had Better EMR than 90% bpEF and Electrically Neutral (50% bpEF) Stimulation Triggered the Fastest Speed

**Figure 5:**
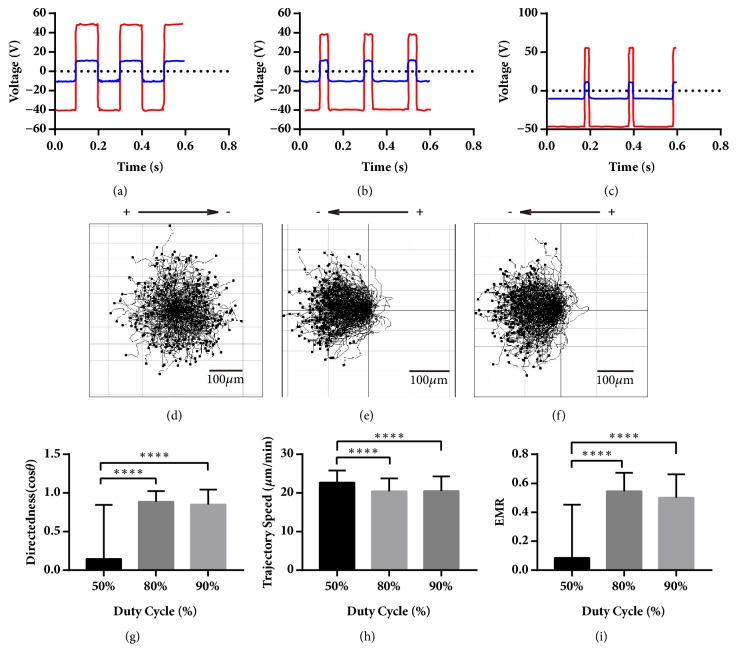
The waveforms of the chamber and the stimulator, the collective galvanotaxis trajectory, and the analysis of the directedness, trajectory speed, and EMR under 5 Hz bpEF with duty cycle 50%, 80%, and 90%. (a-c) The waveforms of the chamber (blue) and the stimulator (red) for 5 Hz bpEF with duty cycle of 50%, 80%, and 90%. (d) The galvanotactic trajectory of all 240 cells under 5 Hz 50% bpEF showed the cells migrated randomly. (e-f) The galvanotactic trajectory of all 240 cells under 5 Hz bpEF of duty cycle 80% (e) and 90% (f) showed that they migrated towards the “net cathodes”. (g) The cells under electrically neutral 5 Hz 50% bpEF were distinct from those under 80% and 90% bpEFs in directedness. ANOVA on different duty cycle: *∗∗∗∗* p < 0.0001. For multiple comparisons, 50% versus 80%: *∗∗∗∗* p < 0.0001; 50% versus 90%: *∗∗∗∗* p < 0.0001. (h) The cells under electrically neutral 5 Hz 50% bpEF migrated the fastest. ANOVA on different duty cycle: *∗∗∗∗* p < 0.0001. For multiple comparisons, 50% versus 80%: *∗∗∗∗* p < 0.0001; 50% versus 90%: *∗∗∗∗* p < 0.0001. (i) The cells under electrically neutral 5 Hz 50% bpEF were distinct from those under 80% and 90% bpEFs in EMR. ANOVA on different duty cycle: *∗∗∗∗* p < 0.0001. For multiple comparisons, 50% versus 80%: *∗∗∗∗* p < 0.0001; 50% versus 90%: *∗∗∗∗* p < 0.0001. One-way ANOVA and Tukey's test of multiple comparisons were used.

The cells were treated with 5 Hz bpEF with duty cycle of 50%, 80%, and 90%. The voltage of the chamber was -10 V in negative stimulation and 10 V in positive that simultaneously established -10 V/cm and 10 V/cm EF in the chamber, respectively; meanwhile the stimulator voltage ranges were -40 to 50 V, -40 to 40 V, and -50 to 50 V, respectively (Figures 5(a), 5(b), and 5(c)). The composition of the periods was 100 ms 10 V/cm EF and 100 ms -10 V/cm EF for 5 Hz 50% duty cycle bpEF, 160 ms and 40 ms for 80% bpEF, and 180 ms and 20 ms for 90% bpEF. The 5 Hz 50% bpEF was electrically neutral, in which the cells migrated randomly towards all directions (Figure 5(d)), with directedness of 0.15 ± 0.70, n = 240, and its mean was approaching 0. The cells under 5 Hz 80% and 90% bpEFs migrated towards the cathode (Figures 5(e) and 5(f)) with directedness of 0.88 ± 0.14, n = 240; 0.85 ± 0.19, n = 240, respectively, and they were distinct (P < 0.0001, P < 0.0001) from 50% duty cycle bpEF (Figure 5(g)). The electrically neutral 50% bpEF triggered the highest (P < 0.0001, P < 0.0001) trajectory speed of 22.67 ± 3.16, n = 240, compared with 20.42 ± 3.37, n = 240, and 20.49 ± 3.83, n = 240, in 80% and 90% bpEFs (Figure 5(h)). For EMR, the 50% bpEF (0.08 ± 0.37, n = 240) was the lowest (P < 0.0001, P < 0.0001), and 80% bpEF (0.55 ± 0.13, n = 240) was not significantly different to 90% bpEF (0.50 ± 0.16, n = 240) except that they showed significant difference in the unpaired* t*-test (Figure 5(i)). In addition, all these 3 parameters of the cells showed significant variation in terms of duty cycle.

**Figure 6:**
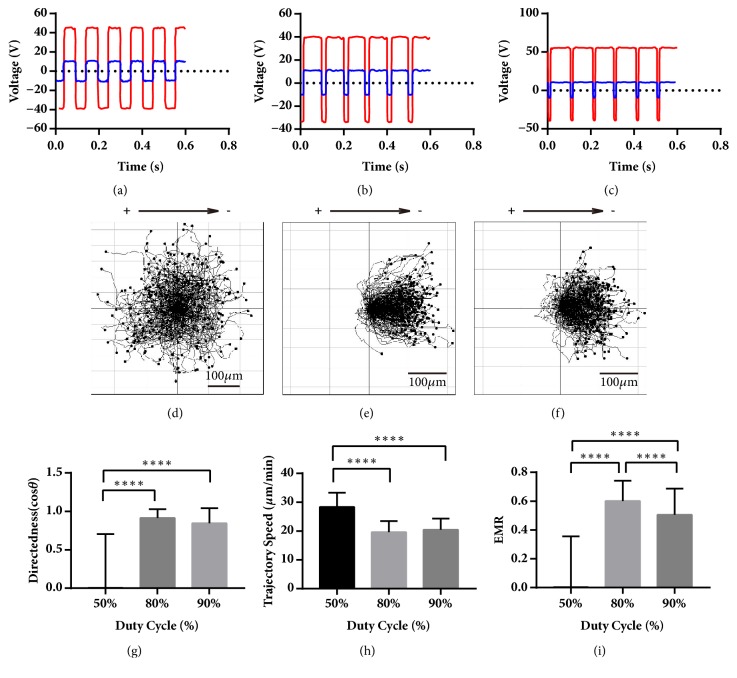
The waveforms of the chamber and the stimulator, the collective galvanotaxis trajectory, and the analysis of the directedness, trajectory speed, and EMR in 10 Hz bpEFs with duty cycle of 50%, 80%, and 90%. (a-c) The waveforms of the chamber (blue) and the stimulator (red) in 10 Hz 50% duty cycle bpEF, 10 Hz 80% bpEF, and 10 Hz 90% bpEF. (d) The galvanotactic trajectory of all 240 cells under 10 Hz 50% bpEF showed that they migrated randomly. (e-f) The galvanotactic trajectory of all 240 cells under 10 Hz bpEF with duty cycle of 80% (e) and 90% (f) showed that they migrated towards the “net cathodes”. (g) The cells under 10 Hz 50% bpEF were distinct from those under 80% and 90% in directedness. ANOVA on different duty cycle: *∗∗∗∗* p < 0.0001. For multiple comparisons, 50% versus 80%: *∗∗∗∗* p < 0.0001; 50% versus 90%: *∗∗∗∗* p < 0.0001. (h) The cells under 10 Hz 50% bpEF migrated the fastest. ANOVA on different duty cycle: *∗∗∗∗* p < 0.0001. For multiple comparisons, 50% versus 80%: *∗∗∗∗* p < 0.0001; 50% versus 90%: *∗∗∗∗* p < 0.0001. (i) The cells under 10 Hz 80% bpEF were the highest in EMR. ANOVA on different duty cycle: *∗∗∗∗* p < 0.0001. For multiple comparisons, 50% versus 80%: *∗∗∗∗* p < 0.0001; 50% versus 90%: *∗∗∗∗* p < 0.0001; 80% versus 90%: *∗∗∗∗* p < 0.0001. One-way ANOVA and Tukey's test of multiple comparisons were used.

The cells were also treated with 10 Hz bpEFs with duty cycle of 50%, 80%, and 90%. The voltage of the chamber was -10 V in negative stimulation and 10 V in positive that simultaneously established -10 V/cm and 10 V/cm EF in the chamber, respectively; meanwhile the stimulator voltage ranges were -40 to 45 V, -35 to 40 V, and -40 to 55 V, respectively (Figures 6(a), 6(b), and 6(c)). The composition of the periods was 50 ms 10 V/cm EF and 50 ms -10 V/cm EF for 50% duty cycle, 80 ms and 20 ms for 80%, and 90 ms and 10 ms for 90%. The cells under 10 Hz 50% bpEF migrated randomly towards all directions (Figure 6(d)), with directedness of 0.004 ± 0.70, n = 240, and its mean was approaching 0. The cells under 10 Hz 80% and 90% bpEFs migrated towards the cathode (Figures 6(e) and 6(f)) with directedness of 0.91 ± 0.12, n = 240, and 0.85 ± 0.20, n = 240, respectively, and they were distinct (P < 0.0001, P < 0.0001) from 10 Hz 50% bpEF (Figure 6(g)). The electrically neutral 10 Hz 50% bpEF triggered the highest (P < 0.0001, P < 0.0001) trajectory speed of 28.31 ± 4.97, n = 240, compared with 19.63 ± 3.80, n = 240, and 20.45 ± 3.86, n = 240, under 10 Hz 80% and 90% bpEFs (Figure 6(h)). For EMR, the 10 Hz 50% bpEF (0.03 ± 0.35, n = 240) was the lowest (P < 0.0001, P < 0.0001), and 10 Hz 80% bpEF (0.60 ± 0.14, n = 240) was higher (P < 0.0001) than 10 Hz 90% bpEF (0.51 ± 0.18, n = 240) (Figure 6(i)). In addition, all these 3 parameters of the cells showed significant variation in terms of duty cycle.

### 3.3. 10 Hz 80% bpEF Induced the Highest Directional Parameters and Electrically Neutral 10 Hz 50% bpEF the Fastest

**Figure 7:**
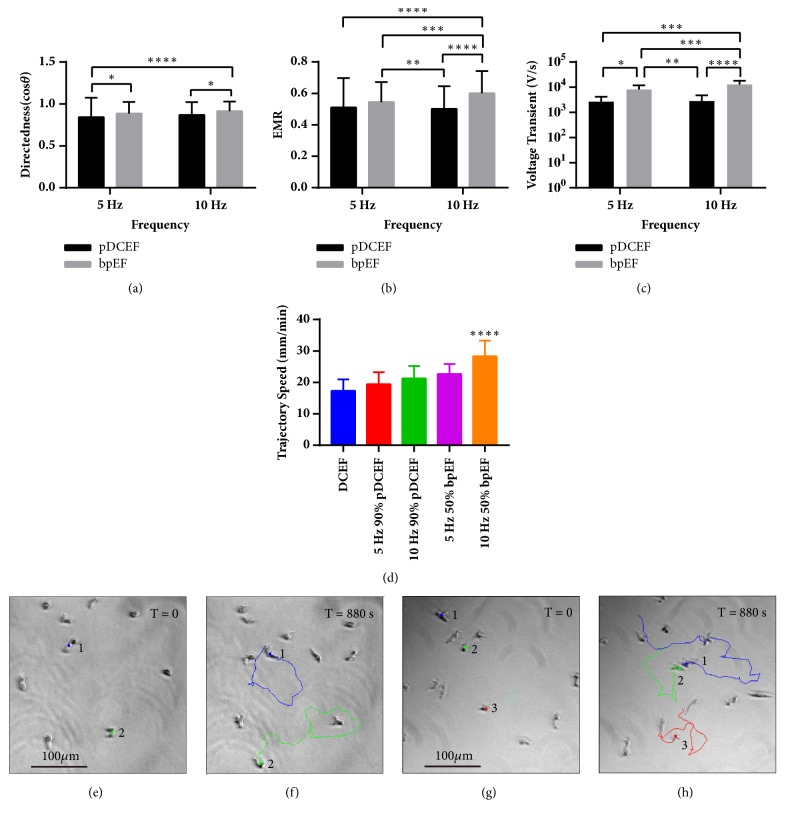
Comparison of directedness and EMR in terms of frequency and polarity and voltage analysis between pDCEFs and bpEFs and comparison of trajectory speed and puzzling circles of the cell in electrically neutral 10 Hz 50% duty cycle bpEF. (a) For directedness, polarity contributed more to statistical variation than frequency, and 10 Hz 80% bpEF induced the highest among all the EFs. Variation on frequency in 2-way ANOVA: *∗* p < 0.05; on polarity: *∗∗∗∗* p < 0.0001; on interaction: ns. For multiple comparisons, 5 Hz pDCEF versus 5 Hz bpEF: *∗* p < 0.05; 5 Hz pDCEF versus 10 Hz bpEF: *∗∗∗∗* p < 0.0001; 10 Hz pDCEF versus 10 Hz bpEF: *∗* p < 0.05. (b) For EMR, 10 Hz 80% bpEF induced the highest among all waveforms. Variation on frequency in 2-way ANOVA: *∗* p < 0.05; on polarity: *∗∗∗∗* p < 0.0001; on interaction: *∗∗∗* p < 0.001. For multiple comparisons, 5 Hz pDCEF versus 10 Hz bpEF: *∗∗∗∗* p < 0.0001; 5 Hz bpEF versus 10 Hz pDCEF: *∗∗* p < 0.01; 5 Hz bpEF versus 10 Hz bpEF: *∗∗∗* p < 0.001; 10 Hz pDCEF versus 10 Hz bpEF: *∗∗∗∗* p < 0.0001. (c) Both the frequency and polarity contributed to statistical variation in voltage transient, and 10 Hz 80% bpEF had the highest voltage transient. Variation on frequency in 2-way ANOVA: *∗* p < 0.05; on polarity: *∗∗∗∗* p < 0.0001; on interaction: *∗* p < 0.05. For multiple comparisons, 5 Hz pDCEF versus 5 Hz bpEF: *∗* p < 0.05; 5 Hz pDCEF versus 10 Hz bpEF: *∗∗∗∗* p < 0.0001; 5 Hz bpEF versus 10 Hz pDCEF: *∗∗* p < 0.01; 5 Hz bpEF versus 10 Hz bpEF: *∗∗∗* p < 0.001; 10 Hz pDCEF versus 10 Hz bpEF: *∗∗∗∗* p < 0.0001. (d) The comparison of trajectory speed of the cells under DCEF, 5 Hz 90% pDCEF, 10 Hz 90% pDCEF, 5 Hz 50% bpEF, and 10 Hz 50% bpEF. ANOVA on different EFs: *∗∗∗∗* p < 0.0001. For multiple comparisons, 5 Hz 50% bpEF versus 10 Hz 50% bpEF: *∗∗∗∗* p < 0.0001; 5 Hz 50% bpEF versus 5 Hz 90% pDCEF: *∗∗∗∗* p < 0.0001; 5 Hz 50% bpEF versus 10 Hz 90% pDCEF: *∗∗* p < 0.01; 5 Hz 50% bpEF versus DCEF: *∗∗∗∗* p < 0.0001; 10 Hz 50% bpEF versus 5 Hz 90% pDCEF: *∗∗∗∗* p < 0.0001; 10 Hz 50% bpEF versus 10 Hz 90% pDCEF: *∗∗∗∗* p < 0.0001; 10 Hz 50% bpEF versus DCEF: *∗∗∗∗* p < 0.0001; 5 Hz 90% pDCEF versus 10 Hz 90% pDCEF: *∗∗∗∗* p < 0.0001; 5 Hz 90% pDCEF versus DCEF: *∗∗∗∗* p < 0.0001; 10 Hz 90% pDCEF versus DCEF: *∗∗∗∗* p < 0.0001. From (e) to (f), cell 1 finished a circular route and cell 2 finished one and a half circles and small curves in 15 mins in the electrically neutral 10 Hz 50% bpEF. From (g) to (h), 3 cells finished acute angle and circle-like routes in 15 mins in electrically neutral 10 Hz 50% bpEF. One-way ANOVA with Tukey's test of multiple comparisons and 2-way ANOVA and Sidak's test of multiple comparisons were used.

To determine the EF with the best directional performance, we compared the directedness and the EMR of the best conditions we had in the abovementioned 4 groups, i.e., 5 Hz 90% pDCEF, 5 Hz 80% bpEF, 10 Hz 90% pDCEF, and 10 Hz 80% bpEF in 2-way ANOVA in terms of frequency and polarity. For directedness (Figure 7(a)), in total, there was significant variation in polarity (P < 0.0001) and frequency (P < 0.05), with no significant variation of interaction (P > 0.05). The cells in bpEFs were higher than pDCEFs in both 5 Hz (P < 0.05) and 10 Hz (P < 0.05). Cells in 10 Hz bpEF migrated towards the cathodes more than that in 5 Hz pDCEF (P < 0.0001). For EMR (Figure 7(b)), in total, there was significant variation in polarity (P < 0.0001), frequency (P < 0.05), and interaction (P < 0.001). Cells in 10 Hz bpEF performed better than those in 5 Hz pDCEF (P < 0.0001), 5 Hz bpEF (P < 0.001), and 10 Hz pDCEF (P < 0.0001). Cells in 5 Hz bpEF performed better than those in 10 Hz pDCEF (P < 0.01).

To determine the highest migration speed, we compared the trajectory speed of the cells in 10 V DCEF, 5 Hz 90% pDCEF, 10 Hz 90% pDCEF, 5 Hz 50% bpEF, and 10 Hz 50% bpEF (Figure 7(d)). The cells under 10 Hz 50% bpEF migrated with the highest trajectory speed (P < 0.0001 for all 4 comparisons) and excluding 10 Hz 50% bpEF, they became higher from 10V DCEF to 5 Hz 50% bpEF (P < 0.0001, P < 0.0001, P < 0.01).

## 4. Discussion

Galvanotaxis is a natural process that exists in almost every species and in some cases overrides other signals in cell migration [39], which is a prerequisite for wound healing, regeneration, and development. The discovery of TEP [5] and TCP [7] opened up the exciting opportunity for researchers to take advantage of this endogenous power [12, 26] for controlling the directional movement of cells using electrical fields. Although predominantly DCEF was used to start with, therapies based on various non-DCEF stimulations were subsequently applied in clinical researches with some promising results [32, 40]. With this new insight and the knowledge that the membrane potential is the foundation of galvanotaxis [41], we sought to compare similarities and differences between DCEF and non-DCEF for stimulating cell migration.

In this study, we applied 10 different types of non-DCEF that are partly adopted from previous clinical studies [32] and 10V/cm DCEF to the cells of* D. discoideum* that is a model organism in developing biology more than 20 years [33] and particularly suitable for chemotaxis and electrotaxis related to wound healing [26, 34]. We then evaluated the cell motility with the parameters of directedness (cos*θ*), trajectory speed (V_cell_), and EMR. The results showed that both pDCEFs and bpEFs were effective in cueing the cells. Furthermore, 90% pDCEF was on par with DCEF in directedness (Figures 3(g) and 4(g)) and EMR (Figures 3(i) and 4(i)) but with greater migration speed (Figures 3(h) and 4(h)) and less power consumption. In terms of directedness (Figure 7(a)) and EMR (Figure 7(b)), 80% bpEF was more effective than pDCEF, which indicates it is also more effective than DCEF. Interestingly, the electrically neutral 10 Hz 50% bpEF induced the fastest movement among all the 10 waveforms (Figure 7(d)), which is partially supported by the previous study that the combination of DCEF and ACEF increased the migration speed of keratinocytes [42]. Additionally, we assume the reason why only 90% pDCEF was better than DCEF in trajectory speed, while 50% pDCEF was lower than DCEF, is that the total net charge in a single period determined the directional characteristics of the cells, but the time-changing polarity of the applied electric field also affected the migration response of the cells due to the electrochemical reason mentioned in the previous study [42]. Although molecular gradients accumulation has been ruled out as a potential trigger in such event, as we previously demonstrated [37], one other possible explanation could be electroosmosis changes triggered by electric pulses, as shown in a recent work by Messerli group [43], which indicates constant DC electric stimuli might attract low molecule substances in the culture medium accumulating and surrounding the migration cells, therefore reducing the cathodal migration response; when paused DCEF was applied, it allowed diffusion of the low molecule substances therefore accelerating the directed migration of the cells. These results suggest that non-DCEF may be more effective in influencing directional ability than DCEF, and electrically neutral bpEF can be used as an alternative therapeutic method in situations where accumulation of the charge should be avoided because the increased trajectory speed could instead contribute to wound healing and regeneration.

In polar EFs, the cells usually move towards cathode and adjust their routes in an obtuse angle, but here we observed circular and U-turn route of the cells in the electrically neutral bpEFs. As shown in Figures 7(e)-7(f) the two cells (cell 1 and 2) located on up-left and down-middle of frame 0 translocated to another position in frame 45 after about 15 mins. During their course of translocation, they completed a circular path which was not observed with the other nonneutral waves. The cells (cells 1, 2, and 3) in Figure 7(g) also translocated in Figure 7(h) in a circular pattern, and further they all had sharp U-turns in their trajectory in the middle of stimulating process, which is comparable with the cells' behavior when the applied DCEF was reversed in a previous study [30]. We assume that there was no retraction of tail and protrusion of new pseudopod in the cells with circular and U-turn movements in the reversal of electric field, as this would have taken the cells several extra minutes. The cells in the neutral waves might have sensed a directional cue, but not sufficient for confirming a uniform direction of movement. The cells which finished U-turns demonstrated to have sensed the reversal of the electric field. The cells that completed circular routes on the other hand appeared to have sensed a changing electric field and responded to it by adjusting their routes. If our assumption of the cells' sense pattern is true, then the basis for galvanotaxis could be cell stimulation by electric pulses, rather than steady currents. In this way, the TEP and TCP can be considered as constant signals and the cells “know” what to do just according to the condition of several critical moments.

One of our main findings here was that the trajectory speed of* D. discoideum* was affected by the voltage of the electric field. This finding does not agree with a previous study by Sato et al. [30], although our experimental conditions differed from them as we used a different voltage control strategy; in our study, all the waveforms applied to the chamber shared the same EF peak – 10 V/cm, and we managed to generate different electric fields by changing the duty cycle and the polarity in a single period rather than directly changing the amplitude of the voltage which is what they did in their study. Additionally, we observed that only the 90% pulsed DCEF was superior to DCEF, while the 50% pulsed DCEF was inferior to it in terms of trajectory speed. We assume that this means repetitive rising edges together with the total net charge in a single period determined the directional characteristics of the cells where the net charge was the main influence factor. This result was also partially supported by a previous study that the combination of DCEF and ACEF increased the migration speed of keratinocytes due to the electrochemical reason [42]. On the other hand, the stronger electroosmotic force on the membrane proteins accumulated towards cathode than anode that polarize the cytoplasmic cell motility machinery to the cathode [43], which seems to be possible reason for this phenomenon. Therefore, an alternative explanation, as shown in a previous study by Messerli group [43], is that constant DCEF might attract low molecule substances in the culture medium accumulating and surrounding the migration cells, therefore reducing the cathodal migration response. We suppose that when paused DCEF or bpEF was applied, it allowed diffusion of the low molecule substances, therefore accelerating the directed migration of the cells.

The parameter of EMR we introduce in this study is very useful for distinguishing cell groups with the same level of directedness but with different trajectory pattern. We can see from Figure 6(g) that, in terms of directedness, the cells in the 80% and 90% duty cycle bpEFs are on the same level, but, in Figures 6(e) and 6(f), we can distinguish them by the overall route angle. The 80% duty cycle bpEF triggered a narrower pattern and an overall straighter route than that of 90% bpEF, and this difference was greater compared with that in directedness (Figure 6(i)). The physical meaning, we comprehend is that, as shown in Figure 1(c), unlike the directedness which compares x-axis component of the displacement of the cells, EMR compares the x-axis component of the displacement of the cell with its actual trajectory. This indicates that EMR is not only dependent upon the origin and terminal points of the cell, but also dependent upon its trajectory and speed, which makes it a suitable parameter for distinguishing between cell groups with the same directedness, but different trajectory patterns, e.g., the different trajectory patterns between 80% and 90% duty cycle (Figures 5(e) and 5(f)), made contribution to the distinction between the EMR of them (Figure 5(i)) while there was little difference between them in directedness (Figure 5(g)).

To test the influence of the reference direction in pDCEFs and bpEFs, we managed to output negative electric waveforms in the first two conditions (Figures 3(a) and 3(b)). The results showed that the reference direction has no influence in directional ability of the cells and suggested that the net charge in a whole period determines the direction followed by the cells. In bpEFs, we applied both positive and negative pulses in a single period (Figures 4(b) and 4(c)). The results reaffirmed the finding that the net charge determines the direction the cell followed (Figures 4(e) and 4(f)). In addition, in neutral bpEFs, no preferred direction was followed by the cells (Figures 5(d) and 6(d)).

When applying bipolar EFs to the cells in the chamber, the important thing to make clear is the direction of the applied EFs. First, we used the duty cycle to specify the direction in this study, as shown in Figures 5 and 6. When the duty cycles were 50% (Figures 5(a) and 6(a)), the EFs were electrically neutral and triggered random migration of the cells (Figures 5(d) and 6(d)). When the duty cycles were not 50% (Figures 5(b) and 5(c); Figures 6(b) and 6(c)), the EFs were polar that induced directional migration of the cells (Figures 5(e) and 5(f); Figures 6(e) and 6(f)). However, it only holds true when the amplitude of negative stimulation was the same as that of the positive one and there was no zero stimulation between them. We think that the better indicator is the average value of the EF used in electrical engineering. Thus, the average EFs of pDCEFs (5 Hz or 10 Hz) with duty cycle of 50% and 90% and bpEFs (5 Hz or 10 Hz) with duty cycle of 50%, 80%, and 90%, for instance, were 5 V/cm, 9 V/cm, 0 V/cm, 6 V/cm, and 8 V/cm, respectively, in contrast to 10 V/cm in DCEF. We assume that, for the EFs with complex voltage waveforms, if their average EFs were 0, the cells might migrate randomly, just as what we observed in 50% bpEFs. On the other hand, the average of the EF did not match the facts that the directedness of the cells under 80% bpEFs were higher than that of 90% bpEFs, and, further, although being with the same average EF, the cells under 10-Hz EFs migrated quicker and more directionally than those in 5-Hz counterparts. We conclude that the frequency, the average, and the polarity of the applied EF all contribute to the migration characteristics of the cells under it.

In an attempt to find the parameters affecting the directional ability of the cells in non-DCEFs, we sampled the voltage transient of 90% duty cycle DCEF and 80% duty cycle bpEF in both 5 Hz and 10 Hz. The voltage transient is defined as 80% of the peak-to-peak voltage divided by its duration, which is the biggest shift of voltage in a single period in each stimulation waveform. In total, there was significant variation in frequency (P < 0.05), polarity (P < 0.0001), and interaction (P < 0.05) of the EF applied. Voltage transient in 10 Hz bpEF was larger than 5 Hz pDCEF (90% duty cycle), 5 Hz bpEF (80% duty cycle), and 10 Hz pDCEF (90% duty cycle) (P < 0.001, P < 0.001, P < 0.0001), respectively, and that of 5 Hz bpEF was larger than pulsed DC in both 5 Hz (P < 0.05) and 10 Hz (P < 0.0001). The results show that, at the same frequency, bpEFs had greater transient rate than pDCEF, and the 10 Hz 80% duty cycle bpEF had the highest voltage transient overall.

The statistical analysis revealed very similar results for voltage transient and that of EMR between these 4 groups (Figure 7(b)). From this finding, we comprehend that the voltage transient, or current pulse, is crucial in determining the directional ability of the cells. On the other hand, voltage transient does not solely determine the directional ability of the cells, because, if so, the cells in 50% duty cycle pDCEF would have the same directional characteristics as in 90%, which is against the data in Figures 3(g) and 3(i). Therefore, through our findings, it may be deduced that duty cycle, the voltage transient, and the frequency are all crucial factors underpinning the directional characteristics of the cells under non-DCEFs.

## 5. Conclusion

In the present investigation, we report that both 80% duty cycle bpEFs and 90% duty cycle pDCEFs induced galvanotaxis of* D. discoideum* with better directional performance than DCEF, with 10 Hz EFs being more effective in stimulating the cells than the 5 Hz ones. The electrically neutral 50% duty cycle bpEFs triggered the highest cell motility with overall random directions. A novel parameter to estimate the directional ability of the cells is introduced here to further discriminate two groups of cells with similar directedness. Electric pulse is found to be relevant to galvanotaxis of the cells. The results show promise for pDCEFs and bpEFs in both basic and clinical research.

## Figures and Tables

**Table 1:** The classification, the waveform types, and time composition of DC, pulsed DC, and bipolar pulse EF stimulation. Uni means Unipolar, i.e., pDCEF; Bip means Bipolar, i.e., bpEF.

Classification	Waveform	Part A (ms)	Part B (ms)	Part C (ms)
DCEF	10 V DC	50	0	0

pDCEF	5 Hz Uni 50%	100	100	0

pDCEF	5 Hz Uni 90%	180	20	0

pDCEF	10 Hz Uni 50%	50	50	0

pDCEF	10 Hz Uni 90%	90	10	0

bpEF	5 Hz Bip 50%	100	0	100

bpEF	5 Hz Bip 80%	160	0	40

bpEF	5 Hz Bip 90%	180	0	20

bpEF	10 Hz Bip 50%	50	0	50

bpEF	10 Hz Bip 80%	80	0	20

bpEF	10 Hz Bip 90%	90	0	10

## Data Availability

The data used to support the findings of this study are available from the corresponding author upon request.
